# Skin Epidermis and Barrier Function

**DOI:** 10.3390/ijms22063035

**Published:** 2021-03-16

**Authors:** Kyung-Min Lim

**Affiliations:** College of Pharmacy, Ewha Womans University, Seodaemungu, Seoul 03760, Korea; kmlim@ewha.ac.kr; Tel.: +82-2-3277-3055; Fax: +82-2-3277-3760

The skin epidermis is the outermost epithelial tissue that protects the body from the external environment. The skin epidermis forms an effective barrier against obnoxious stimuli from outside and simultaneously functions as a semipermeable membrane, helping to maintain proper moisture within the body. The human skin epidermis is relatively thick and robust compared to that of other terrestrial mammals [1], which is most probably due to the paucity of protecting hairs. Even within healthy humans, differences in the structure of skin epidermis and barrier function can be observed [2]. To adapt to the surrounding environment, the skin epidermis has evolved to become a dynamic structure with homeostatic capabilities that can cope with altering external conditions [3,4]. Mostly unidentified complex differentiation/proliferation processes orchestrate the maturation of skin epidermis and its homeostasis.

Disruption of epidermal homeostasis is associated closely with the deterioration of skin health and the pathophysiology of various skin diseases, including ichthyosis, xerosis, atopic dermatitis, and psoriasis, signifying its pathological and aesthetic importance. Consequently, skin epidermis has been an important topic for dermatology, pathology, pharmacology, toxicology, molecular biology, and cosmetic sciences. This Special Issue, “Skin Epidermis and Barrier Function,” comprises excellent articles and reviews related to various aspects of skin epidermis and barrier function at a molecular level and provides important insights into future research on skin epidermis and barrier function.

For such research, a proper test model of human physiology or disease is fundamental. Cunha et al. [5] provided an excellent article introducing human induced pluripotent stem cell (hiPSC)-derived epidermal keratinocytes from ichthyosis patients. These cells can be employed to build human relevant experimental disease models in vitro, which will be of immense help to researching skin diseases. Hwang et al. [6] reported an ex vivo live full-thickness porcine skin model as a versatile tool for skin barrier research and toxicity testing. The ban on animal testing in the cosmetics industry has ignited research on alternative models in dermatology. An ex vivo full-thickness porcine skin model provides such a cheap but human-relevant experimental skin model.

microRNAs (miRNAs) have expanded knowledge on the regulation of mRNA expression in various tissues [7]. Lee [8] provided a comprehensive review of the role of miRNAs in the regulation of epidermal barrier. Beer et al. [9] also reported on the contribution of miR-155 to keratinocyte differentiation and the pathophysiology of psoriasis, highlighting the role of miRNAs in the skin epidermis.

The skin epidermis is an integral topic for cosmetic dermatology since it is important for skin hydration, wound healing, and aging. Rochette et al. [10] provided a comprehensive review on the anti-aging effects of growth differentiation factor 11 (GDF11) in the skin. Choi et al. [11] found that tenascin C (TNC), an element of the extracellular matrix of various tissues, was downregulated in aged skin. More importantly, treatment with recombinant TNC polypeptide increased collagen expression by activating the transforming growth factor- β (TGF-β) signaling pathway. Kobayashi et al. [12] showed that UVB enhances the mis-localization of claudin-1 through NO and peroxynitrite production in a human keratinocyte cell line, which can explain the weakening of skin barrier and the deterioration of skin condition upon exposure to UVB. Lee et al. [13] discovered that a synthetic retinoid, seletinoid G, is effective in improving skin barrier function, which they purported to be due to its wound-healing effects.

Atopic dermatitis is a representative skin disease mainly affecting the epidermis [14]. Souto et al. [15] provided a comprehensive review of treatment strategies for atopic dermatitis. Lee et al. [16] reviewed the relationship of skin barrier abnormalities and immune dysfunction. Lee [17] provided an excellent review purporting the role of skin barrier dysfunction as a pivotal molecular mechanistic cue to induce atopic dermatitis. Toncic et al. [18] discovered that sphingosine, sphinganine, and their ceramides, components of intercellular lipids in the stratum corneum, are altered in atopic dermatitis, which they related to the disrupted skin barrier function of the disease. Ishitsuka and Roop [19] reviewed past and present research on the use of loricrin for skin diseases, based on which they suggested future prospects.

As the guest editor for this Special Issue, I had not expected such enthusiasm from peer researchers. I am grateful to the authors for the contribution of excellent papers to this Special Issue. The second Special Issue under the same title is open for submission now, with a deadline of 31 July 2021. I expect additional excellent articles to be submitted and contribute to knowledge on the skin epidermis and barrier function.

## Data Availability

Not applicable.
